# Regression models for linking patterns of growth to a later outcome: infant growth and childhood overweight

**DOI:** 10.1186/s12874-016-0143-1

**Published:** 2016-04-08

**Authors:** Andrew K. Wills, Bjørn Heine Strand, Kari Glavin, Richard J. Silverwood, Ragnhild Hovengen

**Affiliations:** School of Clinical Sciences & School of Oral & Dental Sciences, University of Bristol, Bristol, UK; Norwegian Institute of Public Health, Oslo, Norway; Diakonova University College, Oslo, Norway; London School of Hygiene and Tropical Medicine, London, UK

**Keywords:** Conditional regression modelling, Growth, Growth patterns, Model parameterisations

## Abstract

**Background:**

Regression models are widely used to link serial measures of anthropometric size or changes in size to a later outcome. Different parameterisations of these models enable one to target different questions about the effect of growth, however, their interpretation can be challenging. Our objective was to formulate and classify several sets of parameterisations by their underlying growth pattern contrast, and to discuss their utility using an expository example.

**Methods:**

We describe and classify five sets of model parameterisations in accordance with their underlying growth pattern contrast (*conditional growth*; *being bigger* v *being smaller*; *becoming bigger and staying bigger*; *growing faster* v *being bigger*; *becoming and staying bigger* versus *being bigger*). The contrasts are estimated by including different sets of repeated measures of size and changes in size in a regression model. We illustrate these models in the setting of linking infant growth (measured on 6 occasions: birth, 6 weeks, 3, 6, 12 and 24 months) in weight-for-height-for-age z-scores to later childhood overweight at 8y using complete cases from the Norwegian Childhood Growth study (*n* = 900).

**Results:**

In our expository example, *conditional growth* during all periods, *becoming bigger* in any interval and *staying bigger* through infancy, and *being bigger* from birth were all associated with higher odds of later overweight. The highest odds of later overweight occurred for individuals who experienced high *conditional growth* or *became bigger* in the 3 to 6 month period and *stayed bigger*, and those who were *bigger* from birth to 24 months. Comparisons between periods and between growth patterns require large sample sizes and need to consider how to scale associations to make comparisons fair; with respect to the latter, we show one approach.

**Conclusion:**

Studies interested in detrimental growth patterns may gain extra insight from reporting several sets of growth pattern contrasts, and hence an approach that incorporates several sets of model parameterisations. Co-efficients from these models require careful interpretation, taking account of the other variables that are conditioned on.

**Electronic supplementary material:**

The online version of this article (doi:10.1186/s12874-016-0143-1) contains supplementary material, which is available to authorized users.

## Background

The global epidemic of childhood obesity is an enormous public health challenge [1]. One area of investigation to help understand how obesity develops is the study of growth. It has been hypothesised that infancy and, in particular, the first six months of life are sensitive windows for later childhood obesity [2–4]. Evidence from observational studies appears compelling. For example, the most recent systematic review reported consistent evidence of positive associations of both infant weight gain from birth to 24 months and size at 5 and 6 months with later body size at 5-13y [5]. However, this review also highlighted the difficulty of comparing results across studies, in part because of the different statistical models used, and concluded that more research is needed to establish whether particular ages are more strongly linked to later body size.

Almost all studies included in the review used a regression model [5]. One difficulty in comparing results is due to the different ways in which each study parameterised the model. For example, some included size at each age while others included changes in size; many studies reported coefficients conditional on past measures of size but a few have also reported coefficients conditional on future changes in size. The interpretation of coefficients from models containing repeated exposures needs to account for the conditioning which is often ignored and can be challenging [6, 7]. For example, a common line of interpretation is: “changes in size in interval X was associated with Y, independent of changes in other intervals” [8]. While correct, such interpretation doesn’t reflect the contrast in growth patterns that is targeted when repeated measures are included in a regression model. To illustrate, the interpretation of the coefficient for birth weight in a model that includes changes in weight from birth to 6 months and 6 to 12 months, compares individuals who were heavier at birth, 6 and 12 months against individuals who were lighter at birth, 6 and 12 months, hence it asks a cumulative question, what is the effect of being bigger from birth to 1y?

Many different growth pattern contrasts can be specified using model parameterisations and re-parameterisations, each targeting a different question. The *conditional growth* parameterisation predominates the growth literature [9], such that investigators often ignore other parameterisations. If the objective is to understand detrimental growth patterns, then given the large variability in growth trajectories among children [10], it seems sensible to implement an analysis strategy that explores the effect of several types of growth pattern, eg; *being bigger* versus *being smaller*, *growing faster* versus *being bigger*. By asking different questions we may get different answers [11], which may provide new insights.

Our objectives were to formulate and describe several model parameterisations to link patterns of infant growth with later childhood overweight, and classify each parameterisation in a way that reflects the underlying growth pattern contrast that is tested. We illustrate the interpretation of these models in an expository analysis using data from the Norwegian Childhood Growth study (NCG) [12]. We aim to describe the utility of this common approach more fully than has been done previously, and draw attention to some of the substantive and statistical issues to consider for future work in this area.

## Methods

### The example dataset and data preparation

The NCG is a national population-based retrospective cohort study of 3180 singleton 3^rd^ grade pupils (mean age 8.3y; range: 7.3 to 9.6y) born in 2002 [12]. Measures of length/height and weight from routine examinations scheduled at birth and at the age of 6 weeks, 3, 6, 9, 12, 15, 18 and 24 months, and 3, 4 and 6 years were extracted from the Norwegian Medical Birth Registry and School records. We use data from birth to 24 months (exposure period) and the 8 year clinic (outcome) as our example. Overweight at the 8y clinic, defined using the age and sex specific International Obesity Task Force criteria (IOTF) [13], was used as the outcome.

The NCG dataset is unbalanced: while there was a target age for each of the routine clinics, some children were measured earlier and some later (see Additional file 1: Figure S1). The regression approach used here requires a balanced or fixed measurement schedule. To adjust each observation to its nearest target age we used a linear interpolation on the z-score scale-each length and weight observation was converted to an age and sex specific z-score using internally generated reference centiles estimated with the LMS (Lambda, Mu, Sigma) curve method [14].

Weight-for-length z-scores at birth, 6 weeks, 3, 6, 12 and 24 months were used as the core set of exposures and calculated using the following equation [15]:

$$ z\left(w{t}_t\Big|le{n}_t\right)=\frac{z\left(w{t}_t\right)-{r}_t.z\left(le{n}_t\right)}{\sqrt{1}-{r}_t^2} $$

where *z*(*wt*_*t*_|*len*_*t*_) is the weight-for-length z-score at target age *t, z*(*wt*_*t*_) and *z*(*len*_*t*_) are the z-scores for weight and length respectively at age *t,* and *r*_*t*_ is the correlation coefficient between weight and height at age *t*. For ease of illustration, we restrict the analysis to the 900 children with complete data.

### Description of models

**Fig. 1:**
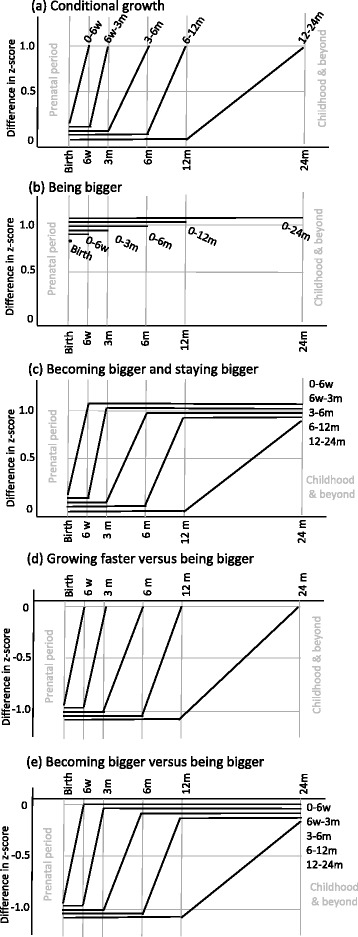
Schematic illustration of the contrasts in growth patterns tested by each of the five models (**a**) to (**e**). The lines plot the difference in weight for length z-score at each age. The thin separation between trajectories is done for clarity; in reality they should be superimposed

We describe five sets of model parameterisations that target five types of growth pattern contrast. The parameterisations are based on incorporating different sets of repeated measures of size and changes in size into the regression equation. Equations (1) to (8) describe the parameterisations and a graphical illustration of the contrast in growth patterns captured by each coefficient is provided in Fig. 1a-e. For pedagogy, the constant term has been removed from the equations below. Likewise, we omit covariables from the example analysis in the results section to make the inter-relations among the different parameterisations clear (see the notes contained in Additional file 1).

#### Growth pattern (a). Conditional growth

Conditional size and conditional growth are the same thing, henceforth we use the term *conditional growth*. These models examine growth in an interval conditional on earlier growth or size. Future size is not conditioned on and so we compare patterns of growth only up to the end of the growth period of interest (Fig. 1a). It thus asks a prospective question at each age: given earlier size, what can we learn about the outcome given current size (or growth in the most recent interval)? The model linking *conditional growth* from birth to 6 weeks to the outcome is:

1 $$ E(Y)={\beta}_0{z}_0+{\beta}_{1.5}{z}_{1.5} $$

where E(Y) is the expected value of the outcome, in our example this is the logit link function for overweight at 8y, and *β*_1.5_ is the regression coefficient for the association between conditional growth from birth to 1.5 months (6 weeks) and the outcome. In our example we use the z-scores *z*_*t*_ at each time point *t* months as the exposures. A sequence of models is thus fitted and the most contemporary coefficient interpreted, capturing growth conditional on the past. By a similar logic to eq. (1), *β*_3_ in eq. (2) below captures the effect of conditional growth from 6 weeks to 3 months:

2 $$ E(Y)={\beta}_0{z}_0+{\beta}_{1.5}{z}_{1.5}+{\beta}_3{z}_3 $$

By construction each period of *conditional growth* is uncorrelated with all other periods, as has been described [9, 11] and shown in Additional file 1: Table S1.

#### Growth pattern (b). Being bigger versus being smaller

There is evidence that childhood obesity may originate in-utero [16], this set of parameterisations draw attention to this by targeting a pattern of *being bigger* versus *being smaller* from birth for different lengths of time through infancy (Fig. 1b). The model to link *being bigger* from birth until 6 weeks with the outcome is:

3 $$ E(Y)={\gamma}_0{z}_0+{\gamma}_{1.5}\left({z}_{1.5}-{z}_0\right) $$

Here, the coefficient for birth size, *γ*_0_, captures the association with being bigger from birth to 6 weeks versus being smaller up to 6 weeks, i.e., it equals the mean difference in the outcome among those one z-score higher at birth controlling for all future changes in size up to 6 weeks. By the same principle, *γ*_0_ in the following model captures the association between being bigger from birth to 3 months of age and the outcome:

4 $$ E(Y)={\gamma}_0{z}_0+{\gamma}_{1.5}\left({z}_{1.5}-{z}_0\right)+{\gamma}_3\left({z}_3-{z}_{1.5}\right) $$

#### Growth pattern (c). Becoming bigger and staying bigger

One possible pathway for overweight is early weight gain that persists through infancy [2]. This parameterisation tries to capture this by examining a pattern of *becoming bigger* in a given period and *staying bigger* through the rest of infancy (Fig. 1c). It thus considers permanent differences in size. The model that captures this contrast is:

5 $$ E(Y)={\delta}_0{z}_0+{\delta}_{1.5}\left({z}_{1.5}-{z}_0\right)+{\delta}_3\left({z}_3-{z}_{1.5}\right)+{\delta}_6\left({z}_6-{z}_3\right) + {\delta}_{12}\left({z}_{12}-{z}_6\right)+{\delta}_{24}\left({z}_{24}-{z}_{12}\right) $$

Where *δ*_1.5_ to *δ*_24_ capture the association of becoming one z-score bigger in each respective interval and maintaining that extra size until 24 months.

#### Growth pattern (d). Growing faster versus being bigger

This set of parameterisations examines the association of change in size in each interval, comparing against children of the same future size (Fig. 1d). It asks the question, among those of the same future size, does starting smaller with subsequent larger increases in size matter? The coefficients in this set of models are actually a test of whether *conditional growth* is any better or worse than *being bigger,* and so we loosely call this set of contrasts *growing faster* versus *being bigger.* The model to test whether *conditional growth* from birth to 6 weeks is any better or worse than *being bigger* from birth to 6w can be written as:

6 $$ E(Y)={\eta}_0\left({z}_{1.5}-{z}_0\right)+{\eta}_{1.5}{z}_{1.5} $$

Here, *η*_0_ is equal to the difference between *β*_1.5_ in eq. (1) (*conditional growth* from birth to 6 weeks) and *γ*_1.5_ in eq. (3) (*being bigger* from birth to 6 weeks), i.e. *η*_0_ = *β*_1.5_ − *γ*_0_ (this can be seen graphically by looking at the contrasts illustrated in Fig. 1a, b & d, and can be shown algebraically - see the online supplementary material). A positive coefficient would mean that *conditional growth* from birth to 6 weeks carries a higher risk of the outcome than *being bigger* from birth to 6 weeks. By the same principle, the coefficient to test for a difference between *conditional growth* from 6w to 3 m and *being bigger* from birth to 3 m is captured by *η*_1.5_ in the following model:

7 $$ E(Y)={\eta}_0\left({z}_{1.5}-{z}_0\right)+{\eta}_{1.5}\left({z}_3-{z}_{1.5}\right)+{\eta}_3{z}_3 $$

#### Growth pattern (e). Becoming and staying bigger versus being bigger

In this model we compare growth patterns of *becoming and staying bigger* through infancy with patterns of *being bigger* from birth throughout infancy (Fig. 1e). It can be estimated using the following:

8 $$ E(Y)={\theta}_0\left({z}_{1.5}-{z}_0\right)+{\theta}_{1.5}\left({z}_3-{z}_{1.5}\right)+{\theta}_3\left({z}_6-{z}_3\right) + {\theta}_6\left({z}_{12}-{z}_6\right)+{\theta}_{12}\left({z}_{24}-{z}_{12}\right) + {\theta}_{24}{z}_{24} $$

where each coefficient for the change in size variables, *θ*_0_ to *θ*_12_, captures the difference between a pattern of *becoming bigger and staying bigger* in each interval versus a pattern of *being bigger.* For example, *θ*_0_ in eq. (8) is equal to *δ*_1.5_ in eq. (5) of the *becoming and staying bigger* model minus *γ*_0_ in the *being bigger* model that includes changes in all intervals up to 24 months (see Fig. 1b, c & e and online supplementary material for proof). A positive coefficient would mean that *becoming bigger* in a period is worse than just *being bigger* from birth.

### Scaling associations across periods

We also investigate the issue of scale which is important to make comparisons across periods and between growth patterns fair. For pedagogical reasons we describe and report these details in the results section.

## Results

### Example using the NCG data

Table 1 shows the results for each of the five models using the NCG data. Growth in weight-for-length during all periods conditional on earlier size (*conditional growth*) was positively associated with later overweight with the largest association occurring in the 3 to 6 m period (OR: 2.1; 95 % CI: 1.5 to 2.9). *Being bigger* in weight for length from birth up to any age in infancy was positively associated with later overweight and the odds were progressively higher the longer the interval of *being bigger* from birth- the odds ratio for later overweight for being one z-score bigger from birth to 24 m was 2.4 (95 % CI: 1.8 to 2.4). A growth pattern of *becoming bigger* in weight for length in any interval and *staying bigger* through infancy was also associated with a higher odds of later overweight. The largest association occurred for gains that persisted from the 3–6 month period (OR: 2.5; 95 % CI: 1.8 to 3.6).

**Table 1:** Odds ratios for overweight per z-score increase in weight for length for each of the five sets of models. The ORs are also adjusted for gestational age and sex

	OR	95 % CI	*p*
(a) Conditional growth:			
Birth to 6w	1.40	1.13, 1.73	0.002
6w to 3 m	1.35	1.00, 1.81	0.049
3 to 6 m	2.07	1.48, 2.89	<0.001
6 to 12 m	1.56	1.15, 2.10	0.004
12 to 24 m	1.53^a^	1.15, 2.02	0.003
(b) Being bigger:			
At birth	1.31	1.10, 1.56	0.003
birth to 6w	1.59	1.28, 1.98	<0.001
birth to 3 m	1.73	1.37, 2.18	<0.001
birth to 6 m	1.95	1.53, 2.49	<0.001
birth to 12 m	2.09	1.63, 2.69	<0.001
birth to 24 m	2.35	1.80, 3.07	<0.001
(c) Becoming bigger and staying bigger:			
Birth to 6w	2.11	1.62, 2.75	<0.001
6w to 3 m	1.87	1.35, 2.59	<0.001
3 to 6 m	2.51	1.77, 3.56	<0.001
6 to 12 m	1.86	1.35, 2.56	<0.001
12 to 24 m	1.53^a^	1.15, 2.02	0.003
(d) Growing faster v being bigger:			
Birth to 6w	0.88	0.72, 1.07	0.19
6w to 3 m	0.78	0.58, 1.06	0.11
3 to 6 m	1.06	0.74, 1.52	0.75
6 to 12 m	0.74	0.53, 1.06	0.099
12 to 24 m	0.65^b^	0.47, 0.90	0.009
(e) Becoming bigger v being bigger:			
Birth to 6w	0.9	0.73, 1.10	0.30
6w to 3 m	0.8	0.58, 1.08	0.15
3 to 6 m	1.07	0.74, 1.53	0.72
6 to 12 m	0.79	0.56, 1.12	0.19
12 to 24 m	0.65^b^	0.47, 0.90	0.009

^a^it is no coincidence that these two coefficients are exactly the same, they are the same contrast (see Fig. 1a & c)

^b^it is no coincidence that these two coefficients are exactly the same, they are the same contrast (see Fig. 1d and e)

In the models that compare *conditional growth* in each interval against patterns of *being bigger*, or put another way, that ask about patterns of starting smaller to become the same size, there was evidence that *conditional growth* from 12 to 24 months was associated with a lower odds of later overweight compared to a pattern of *being bigger* from birth to 24 months. With the exception of the 3 to 6 month period, the results were in the same direction for the other periods, ie, *growing faster* had a lower risk than *being bigger*, but were statistically equivocal. Finally, the results were also equivocal for all intervals in the parameterisation that compared *becoming and staying bigger* against *being bigger* from birth to infancy, or put another way, comparing whether being smaller with later growth in an interval carries a different risk of later overweight to those of the same future size who were bigger at birth. A note on interpretation: the 12–24 month coefficient in this model is the same as the 12–24 month coefficient in the *growing faster* v *being bigger* model because they are the same contrast (Table 1 and Fig. 1d and e).

### Sensitive periods and the issue of scale: using residuals to estimate the growth effects

A common question is whether particular periods of growth or extra size are more strongly linked to the outcome, so called *sensitive periods*. It is useful to think about what we would expect to see in these models if there was a sensitive period. When the outcome is a later version of the exposure and the correlation between measures decreases the further apart in time the exposures are measured, as is the case in our example (see Additional file 1: Table S1), then the closer the exposure is in time to the outcome, the more likely that the prediction will be stronger. This has been called the horse-racing principle [17, 18] - it is easier to pick the winner when the horses are closer to the finish line. For the *conditional growth* and *being bigger* models which ask questions about particular periods but do not condition on the future, we might therefore expect to see a monotonic pattern of stronger associations as the period of growth or extra size gets closer towards the end of infancy. If there is a sensitive period(s) we might expect the coefficient to deviate from this monotonic pattern across periods. For the other models we might expect the coefficients to be similar across periods if there are no sensitive periods.

**Fig. 2:**
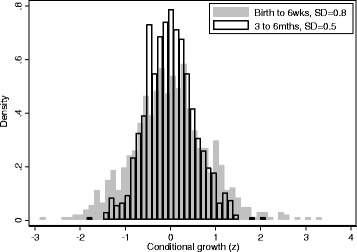
Density histogram of the conditional growth z-scores for the birth to 6 week and 3 to 6 month period

Returning to the NCG data, there was a weak suggestion that growth in the 3 to 6 month interval may be sensitive for later overweight, and in particular, a growth pattern where children *become bigger* from 3 to 6 months and *stay bigger* through infancy – this pattern had the highest OR (Table 1). However, two issues are unaddressed in this analysis. First, formal comparisons between periods lack power due to the sample size, so we cannot exclude sampling error. Second, it is important to try to make comparisons across periods fair. In our example the periods are of unequal duration, so for example, a unit z-score increase between 3 to 6 months is only half the z-score velocity of a unit z-score increase from birth to 6 weeks. Transforming to z-score velocities would resolve this but fails to deal with another potential related issue: a unit increase in velocity may not mean the same thing in each period in population terms because there are periods of growth when population re-ordering or centile crossing is naturally greater. This is illustrated by using residuals and fitting the models in two steps. For example, for the *conditional growth* contrasts, we first estimate each individual’s conditional growth scores by saving the residuals from a series of models regressing size at each age on all earlier sizes. A second analytical model is then fitted regressing the outcome on these residuals (conditional growth scores). Figure 2 overlays the distributions of the conditional growth scores for the birth to 6 week and 3 to 6 month periods in the NCG. The narrower distribution and smaller standard deviation for the 3 to 6 month period (0.8 v 0.5z) implies a period where we expect less conditional growth - a z-score increase in conditional growth in this period thus means 0.8/0.5 = 1.6 times more in population terms than a z-score increase from birth to 6 weeks. A fairer comparison might therefore be to standardise the residuals by dividing by their standard deviation.

The conditional nature of all of the models described here means it is possible to estimate all of the growth contrasts in two steps and hence standardise all of the coefficients in this way. Table 2 describes how to calculate the residuals for the birth to 6 week period in each model, with straightforward extension to other periods.

**Table 2:** Illustration of the models used to estimate the residuals for each of the growth pattern contrasts for the birth to 6 week period. The outcome can then be regressed onto the residuals in a second analytical model^a^

Model	Model to estimate residual for the birth to 6 week period^b^
(a) Conditional growth:	*z*_1.5, *i*_ = *λ*_0_ + *λ*_1_ *z*_0,*i*_ + *ε*_*i*_
(b) Being bigger:	*z*_0, *i*_ = *η*_0_ + *η*_1_(*z*_1.5,*i*_ − *z*_0,*i*_) + *ε*_*i*_
(c) Becoming bigger and staying bigger:	(*z*_1.5, *i*_ − *z*_0, *i*_) = *λ*_0_ + *λ*_1_ *z*_0_ + *λ*_2_(*z*_3_ − *z*_1.5_) + *λ*_3_(*z*_6_ − *z*_3_) + *λ*_4_(*z*_12_ − *z*_6_) + *λ*_5_(*z*_24_ − *z*_12_) + *ε*_*i*_
(d) Growing faster versus being bigger:	(*z*_1.5, *i*_ − *z*_0, *i*_) = *λ*_0_ + *λ*_1_ *z*_1.5_ + *ε*_*i*_
(e) Becoming bigger versus being bigger:	(*z*_1.5, *i*_ − *z*_0, *i*_) = *λ*_0_ + *λ*_1_(*z*_3_ − *z*_1.5_) + *λ*_2_(*z*_6_ − *z*_3_) + *λ*_3_(*z*_12_ − *z*_6_) + *λ*_4_(*z*_24_ − *z*_12_) + *λ*_5_ *z*_24_ + *ε*_*i*_

^a^The residuals *ε*_*i*_ are divided by their standard deviation prior to being entered into the analytical model ie. $\frac{\varepsilon_i}{\mathrm{SD}\left({\varepsilon}_i\right)}$ . In our example the analytical models were also adjusted for sex and gestational age

^b^where *z*_*t*, *i*_ is the z-score for weight for length at age *t* months for subject *i*, and *ε*_*i*_ is the residual for child *i*

Table 3 shows the results incorporating this standardisation. Standardising by the residual standard deviation had the strongest effect on the 3 to 6 month period, drawing the coefficients more towards those for the other periods compared to the unstandardised results. Now the 3 to 6 month period appears less convincing as a sensitive window, although again, the results are equivocal and a larger sample size is required. One aspect to note is that in using this standardisation we lose the tidy algebraic relations where models (d) and (e) can be re-expressed as a sum of models (a) and (b), and (b) and (c) respectively.

**Table 3:** Odds ratios for overweight per z-score increase in weight for length for each of the five sets of models, as estimated using standardised residuals for each of the exposures. The ORs are also adjusted for gestational age and sex

	OR	95 % CI	*p*
(a) Conditional growth:			
Birth to 6w	1.32	1.10, 1.57	0.002
6w to 3 m	1.19	1.00, 1.42	0.049
3 to 6 m	1.48	1.24, 1.78	<0.001
6 to 12 m	1.3	1.09, 1.56	0.004
12 to 24 m	1.32^a^	1.10, 1.58	0.003
(b) Being bigger:			
At birth	1.31	1.10, 1.56	0.003
birth to 6w	1.45	1.22, 1.73	<0.001
birth to 3 m	1.51	1.27, 1.81	<0.001
birth to 6 m	1.64	1.37, 1.97	<0.001
birth to 12 m	1.72	1.43, 2.07	<0.001
birth to 24 m	1.83	1.52, 2.22	<0.001
(c) Becoming bigger and staying bigger:			
Birth to 6w	1.71	1.41, 2.07	<0.001
6w to 3 m	1.42	1.18, 1.71	<0.001
3 to 6 m	1.62	1.35, 1.95	<0.001
6 to 12 m	1.41	1.18, 1.69	<0.001
12 to 24 m	1.32^a^	1.10, 1.58	0.003
(d) Growing faster v being bigger:			
Birth to 6w	0.89	0.75, 1.06	0.19
6w to 3 m	0.87	0.73, 1.03	0.11
3 to 6 m	1.03	0.86, 1.23	0.8
6 to 12 m	0.86	0.72, 1.03	0.01
12 to 24 m	0.79^b^	0.66, 0.94	0.01
(e) Becoming bigger v being bigger:			
Birth to 6w	0.91	0.76, 1.09	0.30
6w to 3 m	0.88	0.74, 1.05	0.14
3 to 6 m	1.03	0.87, 1.23	0.7
6 to 12 m	0.89	0.75, 1.06	0.19
12 to 24 m	0.79^b^	0.66, 0.94	0.01

^a^it is no coincidence that these two coefficients are exactly the same, they are the same contrast (see Fig. 1a & c)

^b^it is no coincidence that these two coefficients are exactly the same, they are the same contrast (see Fig. 1d and e)

## Discussion

We have described five sets of model parameterisations for linking patterns of infant growth with later childhood overweight. The coefficients from these models have a conditional interpretation, and the general approach can be called conditional growth modelling (not to be confused with the *conditional growth* model). The conditioning means that the approach contrasts *growth patterns* or *profiles* rather than *absolute* trajectories. For example, in Fig. 1b the comparison is between patterns of *being bigger* against patterns of *being smaller*, not an absolute trajectory of *being big* versus an absolute trajectory of *being small*.

Most reports of early growth effects have used a form of the *conditional growth* parameterisation. The idea is to compare a child’s growth in an interval against the growth of other children who up until that interval shared a similar growth trajectory, or to compare like for like. We have presented four additional growth pattern contrasts. These models target questions about patterns of *being bigger* from birth up until various ages in infancy, patterns of growth characterised by *becoming bigger and staying bigger* through infancy, and lastly about whether *growing faster* or *becoming bigger* is any better or worse than *being bigger*. While at least two of these parameterisations have been used before, we were unable to find an explicit interpretation of them in terms of the underlying growth pattern contrast [11] and so have tried to classify and offer an interpretation of each in a way in which we feel expresses the substantive research question that each targets.

A debated controversy with this approach concerns how a coefficient in a model conditioned on a repeated measure can be re-expressed to reflect either size or growth. A notable example concerns the developmental origins of health and adult disease hypothesis and the role of birth weight in a model that also conditions on later size [19]. In such a model, a one unit increase in birth weight conditioned on future weight also means a one unit decrease in growth. The question of whether this model implicates size at birth or growth from birth cannot be answered because there is no counterfactual- conditioned on later size, we cannot change earlier size without also changing growth. However, we can interpret the coefficient for birth weight in this model without ambiguity by describing it as a growth pattern contrast, so in this example, it is the association with being born bigger but growing less to become the same future size – this is the inverse of our growth pattern (d) that compares *conditional growth* with *being bigger*. By always interpreting these models with respect to the conditioning as we have attempted, the controversies surrounding re-parameterisations and dual interpretations can be reconciled [11].

Nonetheless, the utility of a model re-parameterisation might be questioned given that the information in the model remains the same, it has just been reshuffled and presented in a different way. Despite this, a re-parameterisation allows a different pattern of growth to be compared and so targets a different question. Reporting and comparing a variety of growth patterns might ensure that important aspects of interpretation are not lost since it is not intuitive to re-express the coefficients from one model to reflect a different growth pattern contrast [11]. Further there is substantial between-child variability in early growth trajectories, and in particular, variability in growth trajectories among children who become obese [10]. A fuller understanding of detrimental growth patterns or in our example, of the origins of childhood obesity in early growth, may thus be better achieved by analyses that investigate several types of pattern. While our example was expository, the wider range of models allowed us to consider associations with a range of growth patterns and put them in context with each other. This meant that attention was not solely focused on intervals of postnatal growth, but also considered patterns where babies are born bigger.

It was not our intention to prescribe a framework of parameterisations for future analyses; the models we present are just one of many possible sets of parameterisations for exploring growth. For example, interactions between periods and the notion of catch up growth among smaller babies, the association of being bigger over different intervals of infancy instead of just from birth, and being bigger conditional on past and future size [20] could also be parameterised, along with non-linear relationships and questions about whether there is a threshold of weight gain that is particularly detrimental.

Multilevel models (MLMs) are widely used in studies of growth effects. The approach involves fitting an MLM to estimate each individual’s growth trajectory as a function of age (growth model), then regressing the outcome on the MLM-based predicted individual-level values (analytical model). They have the advantage of dealing, to some extent, with several of the statistical issues inherent to analyses of growth data such as missing data, irregular measurement schedules and measurement error. Often however, the analytical model is then parameterised as a *conditional growth* model [21, 22], and so asks the same substantive question about *conditional growth*. Nonetheless, other parameterisations, such as those here, could be formulated within an approach that uses an MLM as a first step.

Non-linear models can offer a different type of parameterisation and thus ask a different question. For example, the SITAR model [23, 24] describes a trajectory using three parameters that correspond to size, velocity and developmental tempo. A unique feature of the SITAR model is that by providing a parameter for the age scale it acknowledges that individuals grow on different developmental trajectories and thus allows developmental features such as age at adiposity rebound to vary across individuals. Latent growth models also offer a substantively different comparison. These models group individuals based on their underlying (latent) trajectory and so compare absolute rather than conditional trajectories. Latent class models are data driven - the comparison is derived from the data rather than designed by the analyst, so comparisons may not capture any a priori research question. Much may be gained by considering a framework of different approaches and models [6].

When the interest is in sensitive periods, it is important to consider how to make comparisons across periods fair. The use of z-scores of size and/or z-score velocities will not account for any differences in the level of population re-ordering or centile crossing in each period. Using standardised residuals as illustrated here can control for this. However, it is important to think about whether this sort of standardisation is appropriate as it may affect the conclusions drawn from the study. Another aspect which may warrant consideration is measurement error, which is likely to depend on age, and therefore could bias comparisons between periods.

Studies interested in windows of growth require a sample size and measurement schedule that allow periods of interest to be examined with sufficient power and minimal bias. Missing data can reduce power and cause bias. In our example, the sample size was substantially reduced when restricting to complete cases. Several options exist for dealing with missing data in a more principled way, for example, modelling individual trajectories as a first step using an MLM, or formulating the models in a structural equation or path analysis framework then using full information maximum likelihood to estimate the parameters. There is no good reason to only work with the complete cases except for simplicity (as we did in this expository example). Lastly, larger samples are also needed for narrower time windows because of the effects of model collinearity, measurements close in time tend to be more correlated and this will cause larger standard errors.

## Conclusions

Much attention has duly been given to the statistical issues that arise in the analysis of growth exposures; while clearly important, it is also prudent to consider the substantive question(s) that underpin the model(s) adopted. As we have shown, different parameterisations of a regression model offer one way of targeting other substantive questions concerning growth. Our interest was in the utility of re-parameterisations in this setting, alternative questions about growth to those we have described are likely to be asked, and will require different parameterisations and input from experts in other fields. Nonetheless, the general approach of considering a range of parameterisations may have added utility for research into early growth and later outcomes above and beyond an analysis that only considers the classic *conditional growth* model.

### Ethics approval & consent to participate

The regional Committee for Medical Research Ethics approved the study that was used as an example in this paper. Written consent for participation, retrieval of data from the Medical Birth Registry and from the well-child clinic health records was obtained from one parent of each participant.

### Availability of data and materials

Enquiries about access to data from the Norwegian Childhood Growth study can be made to the Norwegian Institute of Public Health, Oslo, Norway.

## Additional file

Additional file 1: Table S1. Correlation among measures of weight for length size, changes in weight for length and conditional growth in weight for length (n=900) (all growth z-scores). Also shown are the unconditional (unadjusted) odds ratios for overweight at 8years. **Figure S1.** Scatter of raw data and internally generated growth centiles (estimated using the LMS method) for length/height and BMI in the NCG cohort. Boys are on the right and girls on the left. Also shown is the mean WHO z-score at each appointment. The coloured scatter and red line represent those individuals included in the illustrative analysis (complete cases), the grey scatter and black WHO z-score are those excluded due to some missing data. (DOCX 276 kb)

## Abbreviations

BMI: body mass index
CI: confidence interval
IOTF: International Obesity Task Force
LMS: Lambda, mu, sigma
MLM: multilevel models
NCG: Norwegian Childhood Growth study
OR: odds ratio
