# Supplementary material for: Regression models for linking patterns of growth to a later outcome: infant growth and childhood overweight
Source: BMC Med Res Methodol. 2016 Apr 8;16:41. doi: 10.1186/s12874-016-0143-1 (PMC4826511; doi:10.1186/s12874-016-0143-1)
Supplement: Additional file 1: Table S1. — Correlation among measures of weight for length size, changes in weight for length and conditional growth in weight for length (n=900) (all growth z-scores). Also shown are the unconditional (unadjusted) odds ratios for overweight at 8years. Figure S1. Scatter of raw data and internally generated growth centiles (estimated using the LMS method) for length/height and BMI in the NCG cohort. Boys are on the right and girls on the left. Also shown is the mean WHO z-score at each appointment. The coloured scatter and red line represent those individuals included in the illustrative analysis (complete cases), the grey scatter and black WHO z-score are those excluded due to some missing data. (DOCX 276 kb) [file 12874_2016_143_MOESM1_ESM.docx]

**Online supplementary material**

Table S1. Correlation among measures of weight for length size, changes in weight for length and conditional growth in weight for length (n=900) (all growth z-scores). Also shown are the unconditional (unadjusted) odds ratios for overweight at 8years.

|  |  | Size | | | | | | Change in size | | | | | Conditional growth | | | | |
| --- | --- | --- | --- | --- | --- | --- | --- | --- | --- | --- | --- | --- | --- | --- | --- | --- | --- |
|  |  | Birth | 6w | 3m | 6m | 12m | 24m | 0 to 6w | 6w to 3m | 3 to 6m | 6 to 12m | 12 to 24m | 0 to 6w | 6w to 3m | 3 to 6m | 6 to 12m | 12 to 24m |
| Size | Birth | 1 |  |  |  |  |  |  |  |  |  |  |  |  |  |  |  |
|  | 6w | 0.43 | 1 |  |  |  |  |  |  |  |  |  |  |  |  |  |  |
|  | 3m | 0.26 | 0.76 | 1 |  |  |  |  |  |  |  |  |  |  |  |  |  |
|  | 6m | 0.21 | 0.62 | 0.83 | 1 |  |  |  |  |  |  |  |  |  |  |  |  |
|  | 12m | 0.23 | 0.51 | 0.65 | 0.79 | 1 |  |  |  |  |  |  |  |  |  |  |  |
|  | 24m | 0.22 | 0.40 | 0.52 | 0.63 | 0.73 | 1 |  |  |  |  |  |  |  |  |  |  |
| Change in size | 0 to 6w | -0.59 | 0.47 | 0.44 | 0.35 | 0.23 | 0.15 | 1 |  |  |  |  |  |  |  |  |  |
|  | 6w to 3m | -0.25 | -0.32 | 0.37 | 0.32 | 0.21 | 0.18 | -0.04 | 1 |  |  |  |  |  |  |  |  |
|  | 3 to 6m | -0.06 | -0.21 | -0.25 | 0.34 | 0.29 | 0.22 | -0.13 | -0.05 | 1 |  |  |  |  |  |  |  |
|  | 6 to 12m | 0.04 | -0.14 | -0.25 | -0.28 | 0.36 | 0.18 | -0.17 | -0.16 | -0.07 | 1 |  |  |  |  |  |  |
|  | 12 to 24m | -0.03 | -0.16 | -0.19 | -0.25 | -0.40 | 0.33 | -0.11 | -0.05 | -0.10 | -0.26 | 1 |  |  |  |  |  |
| Conditional growth | 0 to 6w | 0.00 | 0.90 | 0.73 | 0.59 | 0.45 | 0.35 | 0.81 | -0.23 | -0.20 | -0.18 | -0.16 | 1 |  |  |  |  |
|  | 6w to 3m | 0.00 | 0.00 | 0.64 | 0.54 | 0.40 | 0.34 | 0.00 | 0.94 | -0.13 | -0.20 | -0.11 | 0.00 | 1 |  |  |  |
|  | 3 to 6m | 0.00 | 0.00 | 0.00 | 0.56 | 0.47 | 0.36 | 0.00 | 0.00 | 0.97 | -0.12 | -0.16 | 0.00 | 0.00 | 1 |  |  |
|  | 6 to 12m | 0.00 | 0.00 | 0.00 | 0.00 | 0.60 | 0.36 | 0.00 | 0.00 | 0.00 | 0.95 | -0.34 | 0.00 | 0.00 | 0.00 | 1 |  |
|  | 12 to 24m | 0.00 | 0.00 | 0.00 | 0.00 | 0.00 | 0.68 | 0.00 | 0.00 | 0.00 | 0.00 | 0.90 | 0.00 | 0.00 | 0.00 | 0.00 | 1 |
|  |  |  |  |  |  |  |  |  |  |  |  |  |  |  |  |  |  |
| Overweight at 8y | Odds ratio | 1.29 | 1.47 | 1.49 | 1.75 | 1.83 | 1.96 | 1.06 | 1.06 | 1.66 | 1.19 | 1.03 | 1.40 | 1.33 | 2.04 | 1.52 | 1.48 |
|  | Lower CI | 1.09 | 1.21 | 1.24 | 1.45 | 1.52 | 1.61 | 0.89 | 0.81 | 1.21 | 0.91 | 0.80 | 1.13 | 0.99 | 1.46 | 1.14 | 1.12 |
|  | Upper CI | 1.54 | 1.78 | 1.81 | 2.12 | 2.21 | 2.40 | 1.26 | 1.40 | 2.28 | 1.58 | 1.31 | 1.73 | 1.78 | 2.83 | 2.04 | 1.95 |
|  | p-value | 0.00 | 0.00 | 0.00 | 0.00 | 0.00 | 0.00 | 0.49 | 0.67 | 0.00 | 0.21 | 0.83 | 0.00 | 0.06 | 0.00 | 0.00 | 0.01 |


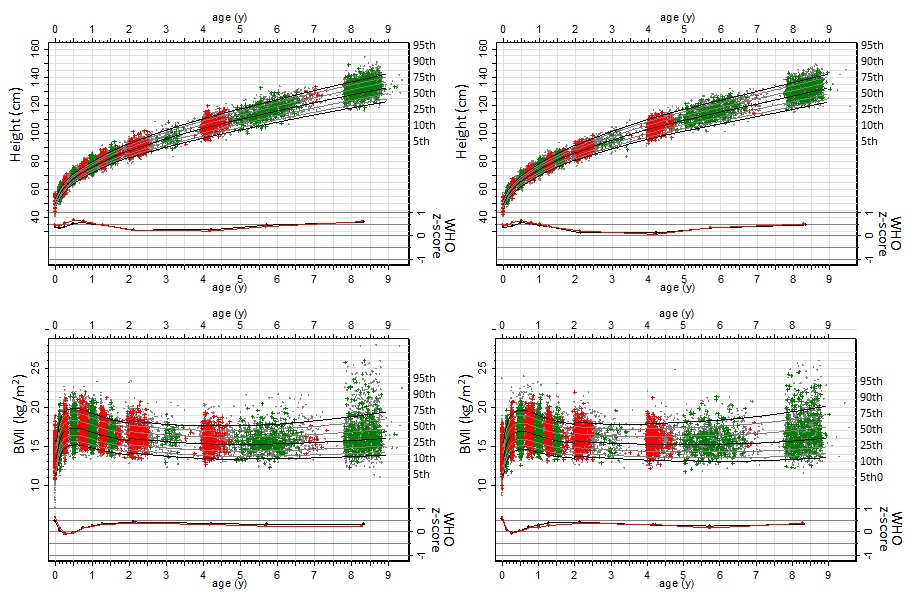


Figure S1. Scatter of raw data and internally generated growth centiles (estimated using the LMS method) for length/height and BMI in the CGS cohort. Boys are on the right and girls on the left. Also shown is the mean WHO z-score at each appointment. The coloured scatter and red line represent those individuals included in the illustrative analysis (complete cases), the grey scatter and black WHO z-score are those excluded due to some missing data.

Some notes concerning the re-expression of the model parameterisations in the main text

In the main text of our paper we state that the *growing faster versus being bigger* model (model d) can be used to test whether *conditional growth* (model a) is any better or worse than *being bigger (model b)*. To show this, consider the simplest setting with an exposure $X_{t}$ measured at 2 time points, *t=1,2*.

The *conditional growth* model (equation 1 in the main text) can be written as

$E\left( Y \right)=\beta_{0}+\beta_{1}X_{1}+\beta_{2}X_{2}$ [a]

Where $\beta_{2}$ captures the effect of *conditional growth* in *X* from *t1* to *t2*.

The *being bigger* model (equation 3 in the main text) can be estimated using:

$E\left( Y \right)=\gamma_{0}+\gamma_{1}X_{1}+\gamma_{2}(X_{2}-X_{1})$ [b]

Where $\gamma_{1}$ captures the effect of being bigger at t1 and t2.

The *growing faster versus being bigger* model (equation 6 in the main text) can be written as:

$E\left( Y \right)=\eta_{0}+\eta_{1}(X_{2}-X_{1})+\eta_{2}X_{2}$ [c]

As we stated in the main text of the paper, $\eta_{1}$ captures the difference between *conditional growth* from *t1* to *t2* and *being bigger* from t1 to t2, i.e:

$\eta_{1}=\beta_{2}-\gamma_{1}$ [d]

To show this, first consider that equations [a] & [b] are re-parameterisations of each other -expanding the brackets in equation [b] and collecting coefficients for $X_{1}$ and $X_{2}$ shows that $\gamma_{2}$ in the being bigger model is also a measure of conditional growth from t1 to t2 or $\gamma_{2}$=$\beta_{2}$.

$E\left( Y \right)=\gamma_{0}+{(\gamma}_{1}-{\gamma_{2})X}_{1}+\gamma_{2}X_{2}$ [e]

Therefore [d] can also be written as:

$\eta_{1}=\gamma_{2}-\gamma_{1}$ [f]

Then, similarly expanding the *growing faster versus being bigger* model [c] and collecting coefficients for $X_{1}$ and $X_{2}$ gives:

$E\left( Y \right)=\eta_{0}-\eta_{1}X_{1}+{(\eta}_{1}+\eta_{2}) X_{2}$ [g]

comparing [g] and [e] it follows that:

-$\eta_{1}=\gamma_{1}-\gamma_{2}$ [h]

And hence shows how $\eta_{1}$ in [3] is equal to a test of $\beta_{2}-\gamma_{1}$ or $\gamma_{2}-\gamma_{1}$

This illustration also shows how the coefficients in these 3 models are re-parameterisations of each other which means that we could actually estimate only one model and derive the estimates of all of the other growth pattern contrasts from that using linear combinations of the coefficients. There are also several other ways each of these growth pattern contrasts could be estimated. In the paper we showed how to estimate the 3 growth pattern contrasts using 3 different parameterisations, one for each growth pattern contrast; we did this because we felt it more explicitly links the models back to the underlying research question.

In the paper we also stated that the coefficients, $\theta_{0}$ to $\theta_{12}$, in the *becoming bigger model v being bigger* (model e) capture the difference between *becoming bigger* in each period and *staying bigger* against merely *being bigger* through infancy. We can show this again in the simplest setting possible by considering an exposure $X_{t}$ measured at 3 time points, *t=1 to 3*.

The *being bigger* from t=1 to t=3 model is written as:

$E\left( Y \right)=\gamma_{0}+\gamma_{1}X_{1}+\gamma_{2}\left( X_{2}-X_{1} \right)+\gamma_{3}(X_{3}-X_{2})$ [i]

Where $\gamma_{1}$ is interpreted as the association between being a unit bigger from t=1 to t=3.

The *becoming bigger and staying bigger* until *t=3* model is actually the same as equation [i] but for completeness and keeping the same $\delta$ notation as in the paper, it is:

$E\left( Y \right)=\delta_{0}+\delta_{1}X_{1}+\delta_{2}\left( X_{2}-X_{1} \right)+\delta_{3}(X_{3}-X_{2})$ [j]

Where $\delta_{2}$ captures the association between *becoming bigger* from t1 to t2 and *staying bigger* until t3.

The *becoming bigger versus being bigger* model for our simple scenario can be written as:

$E\left( Y \right)=\theta_{0}+\theta_{1}\left( X_{2}-X_{1} \right)+\theta_{2}\left( X_{3}-X_{2} \right)+\theta_{3}X_{3}$ [k]

Where $\theta_{1}$ captures the difference between *becoming bigger* from t1 to t2 and *staying bigger* until t3 versus *being bigger* from t1 to t3, i.e;

$\theta_{1}=\delta_{2}- \gamma_{1}$ = $\delta_{2}- \delta_{1}$ [l]

This can be shown by expanding equations [j] and [k] and collecting the coefficients for $X_{1} to X_{3}$:

$E\left( Y \right)=\delta_{0}+{(\delta}_{1}{-\delta_{2})X}_{1}+{(\delta}_{2}-\delta_{3})X_{2}+\delta_{3}X_{3}$ [m]

$E\left( Y \right)=\theta_{0}-{\theta_{1}X}_{1}+{(\theta}_{1}-\theta_{2})X_{2}+{(\theta}_{2}+\theta_{3})X_{3}$ [n]

Comparing [m] and [n] gives [l]; i.e, ${-\theta}_{1}= \delta_{1}-\delta_{2}$ or $\theta_{1}= \delta_{2}-\delta_{1}$
